# 2490. Improving Hepatitis C Continuum of Care in an Outpatient Internal Medicine Population

**DOI:** 10.1093/ofid/ofad500.2108

**Published:** 2023-11-27

**Authors:** Deepali Boothankad Sharath, Timothy McCann, Neha Patel, Sarthak Aryal, Usha Thapa, Cynthia Contreras, Anar Patel

**Affiliations:** TriHealth Good Samaritan Hospital, Cincinnati, Ohio; TriHealth Good Samaritan Hospital, Cincinnati, Ohio; TriHealth Good Samaritan Hospital, Cincinnati, Ohio; TriHealth Good Samaritan Hospital, Cincinnati, Ohio; TriHealth Good Samaritan Hospital, Cincinnati, Ohio; TriHealth Good Samaritan Hospital, Cincinnati, Ohio; TriHealth Infectious Diseases, Cincinnati, Ohio

## Abstract

**Background:**

According to the WHO, approximately 71 million people are infected with Hepatitis C virus (HCV), and more than 400,000 people die each year due to HCV-related liver diseases. Since the advent of safe and highly effective direct-acting antivirals (DAA) in 2014, treatment for HCV has been revolutionized with cure rates exceeding 90%. Although the detection of undiagnosed HCV infection itself likely has some inherent benefit, the impact of screening is greatly reduced if it does not lead to treatment and cure. Studies on the HCV continuum of care have concluded many patients are lost at each stage, highlighting the need to raise awareness among healthcare professionals and at-risk populations about hepatitis testing, referral, support, and care. A practical model to help fill care gaps in urban internal medicine clinics and improved treatment enrollment and adherence is necessary, which in turn will improve public health outcomes.

**Methods:**

The objective of the study is to improve the continuum of care of HCV, from diagnosis to treatment of the disease at the outpatient Faculty Medical Center (FMC) Internal Medicine Clinic, and identify hurdles to treatment. We enrolled 31 patients who screened positive for hepatitis C antibody into a pooled cohort, where biweekly telephone encounters were initiated for 3 months to facilitate close follow-up, including scheduling labs and office visits and facilitating subspecialty referrals. (Table 1).

Hepatitis C Continuum of Care Workflow

**Figure:**
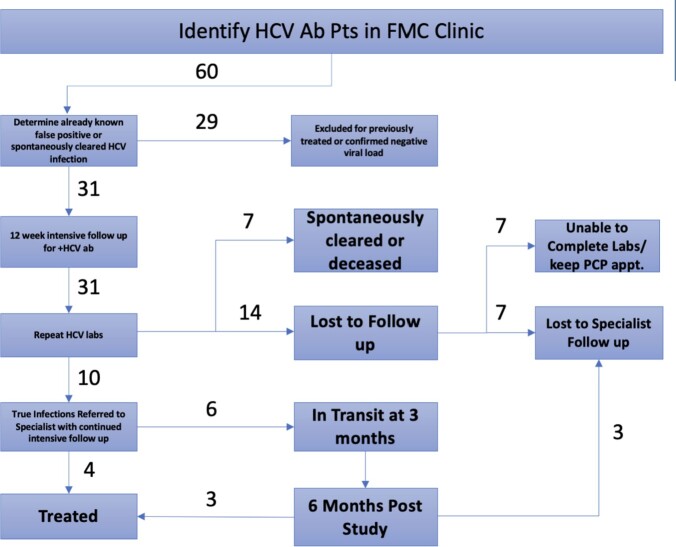

HCV labs include HCV viral load and genotype, Fibroscan, Hepatitis B surface antigen, and antibody, Hepatitis A antibody, and HIV 1/2 antibody test.

**Results:**

Six months post-study, 7 out of 31 (22%) patients enrolled in our intensive follow-up completed treatment. Among the patients who were lost to follow-up, the most common issue encountered was following up with specialists (58%). The most common communication barrier was patients having inactive phone numbers.

Results

**Figure:**
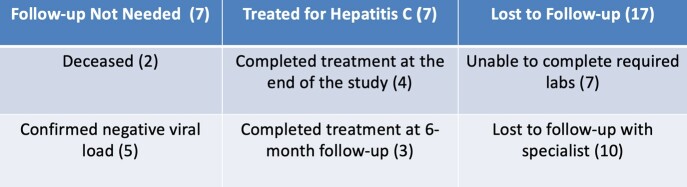

**Conclusion:**

Our next step is to establish an HCV clinic within our FMC Internal Medicine Center to expand access to care for HCV-affected patients. The HCV clinic will include coordination with the Infectious Diseases specialist through E-Consult and TriHealth Specialty Pharmacy. The impact will be early treatment of HCV-affected patients, improved treatment success which ultimately reduces the risk of transmission and progression of the disease, and improved quality of life.

**Disclosures:**

**All Authors**: No reported disclosures

